# Genome wide noninvasive prenatal testing detects microduplication of the distal end of chromosome 15 in a fetus: a case report

**DOI:** 10.1186/s13039-022-00592-3

**Published:** 2022-04-02

**Authors:** Hana Sahinbegovic, Stephanie Andres, Sabine Langer-Freitag, Aspasia Divane, Fotini Ieremiadou, Senad Mehmedbasic, Aida Catic

**Affiliations:** 1 Department of Clinical Studies, University of Ostrava, 70300 Ostrava, Czech Republic; 2 Institute of Human Genetics, Klinikum Rechts Der Isar, Technical University of Munich, 81675 Munich, Germany; 3 LIFE CODE Private Diagnostic Laboratory, Medical Ltd., 11523 Athens, Greece; 4 Institute for Gynecology, Perinatology, and Infertility “Mehmedbašić”, 71000 Sarajevo, Bosnia and Herzegovina

**Keywords:** Noninvasive prenatal test, 15q26.1-qter partial trisomy, Microduplication, Prenatal testing

## Abstract

**Background:**

Noninvasive prenatal testing (NIPT) is the most recent modality widely used in prenatal diagnostics. Commercially available NIPT has high sensitivity and specificity for the common fetal chromosomal aneuploidies. As future advancements in NIPT sequencing technology are becoming promising and more reliable, the ability to detect beyond aneuploidies and to expand detection of submicroscopic genomic alterations, as well as single-gene disorders might become possible.

**Case presentation:**

Here we present a case of a 34-year-old pregnant woman, G2P1, who had NIPT screening which detected a terminal microduplication of 10.34 Mb on the long arm of chromosome 15 (15q26.1q26.3). Subsequent prenatal diagnostic testing including karyotype, microarray and fluorescence in situ hybridization (FISH) analyses were performed. Microarray testing confirmed and particularized a copy number gain of 10.66 Mb of the distal end of the long arm of chromosome 15. The G-banding cytogenetic studies yielded results consistent with unbalanced translocation between chromosome 15 and 18. To further characterize the abnormality involving the long arm of chromosome 18 and to map the genomic location of the duplicated 15q more precisely, FISH analysis using specific sub-telomeric probes was performed. FISH analysis confirmed that the extra duplicated segment of chromosome 15 is translocated onto the distal end of the long arm of chromosome 18 at band 18q23. Parental karyotype and FISH studies were performed to see if this unbalanced rearrangement was inherited from a healthy balanced translocation carrier versus being a de novo finding. Parental chromosomal analysis provided no evidence of a rearrangement between chromosome 15 and chromosome 18. The final fetal karyotype was reported as 46,XX,der(18)t(15;18)(q26.2;q23)dn.

**Conclusions:**

In this case study, the microduplication of fetal chromosome 15q26.1q26.3 was accurately detected using NIPT. Our results suggest that further refinements in NIPT have the potential to evolve to a powerful and efficient screening method, which might be used to detect a broad range of chromosomal imbalances. Since microduplications and microdeletions are a potential reportable result with NIPT, this must be included in pre-test counseling. Prenatal diagnostic testing of such findings is strongly recommended.

## Introduction

The use of maternal serum marker screening and ultrasound imaging (ultrasonography) to detect chromosome aneuploidies and other birth defects are traditional approaches of prenatal care in the first and/or second trimesters [1]. If these tests indicate that a fetus is at an increased risk for a genetic disorder, invasive methods such as chorionic villus sampling (CVS) or amniocentesis are recommended for diagnostic testing. More recently, large systemic review reported significantly lower procedure related risks of pregnancy complications, including miscarriage [2]. The use of non-invasive prenatal screening test (NIPT) has grown rapidly, leading to a simultaneous reduction in the application of traditional analyte screening tests and invasive diagnostic procedures [3].

Clinical translation of NIPT technologies has revolutionized prenatal care. NIPT measures and evaluates small fragments of placental DNA that are circulating in a pregnant woman’s blood. The cell-free DNA (cfDNA) in maternal plasma reflects the genetic makeup of the developing fetus. Commercially available NIPT is used as a prenatal screening technique allowing for detection of the most common autosomal and sex chromosome aneuploidies. Further advances in NIPT technology have shown that micropulications and microdeletions can be detected. Many professional societies currently recommend that NIPT be used as a screening tool, not a definitive diagnostic test; therefore, when cell-free DNA results are at high risk, the diagnosis requires subsequent follow-up testing by means of genetic analysis of samples collected invasively. NIPT provides high sensitivity (true positive rate) and specificity (true negative rate), making it an attractive alternative to the serum screens and invasive diagnostics currently in use [4, 5].

A normal human cell is made up of 46 chromosomes that are grouped into 24 different types and arranged in 23 homologous pairs. Of those 23 pairs, 22 are autosomes, and the remaining pair is comprised of the two different types of two sex chromosomes, which specify gender (XX for female and XY for male) [6]. Each chromosomal homologous pair consists of one maternal and one paternal chromosome that pair up with each other inside a cell during meiosis [7]. Chromosomal disorders mostly fall into two main categories: numerical and structural abnormalities [8]. Chromosomal anomalies or aneuploidies, represented primarily by numerical change, are the single greatest contributor to prenatal morbidity and mortality [9]. Karyotyping, fluorescence in situ hybridization (FISH), quantitative fluorescence polymerase chain reaction (QF-PCR), chromosomal microarray (CMA), and the next-generation sequencing (NGS) are the common methods used for prenatal diagnostics [10].

Herein, we present a clinical prenatal case in which NIPT testing detected a 10.34 Mb gain (duplication) of the long arm of chromosome 15. Clinical value of NIPT finding was further confirmed by microarray analysis in conjunction with karyotyping and FISH analysis.

## Clinical course

A 34-year-old pregnant woman, G2P1, gestational age 10 weeks and 5 days, came to prenatal clinic for routine prenatal care. Patient elected to screen for the common fetal chromosomal aneuploidies using NIFTY standard panel. Routine first trimester ultrasound examination did not indicate any fetal abnormalities. No history of spontaneous abortions was reported. Family history was remarkable, no chromosomal anomalies or other genetic disorders were reported.

## Materials and methods

### Noninvasive prenatal screening

Noninvasive screening test was performed at NIFTY (powered by Geneplanet, Ljubljana, Slovenia). A blood sample required (minimum of 10 ml) for NIPT testing was drawn at > 10 weeks of gestation. NIFTY standard panel screens for the common autosomal aneuploidies and sex chromosome aneuploidies.

### Genomic microarray analysis

Genomic microarray analysis on isolated DNA from amniotic fluid cells was performed at Life Code Laboratories (Athens, Greece). Briefly, genomic DNA from amniotic fluid was extracted using NucleoSpin blood extraction kit by Macherey–Nagel. CytoScan® Optima Array by Affymetrix (Santa Clara, CA, USA) was used for the detection of copy number variations (CNV) and loss of heterozygosity (LOH), according to manufacturer’s instructions. Results were analyzed using Chromosome Analysis Suite (ChAS). Additional databases referenced for the analysis included: Decipher, DGV, ClinVar, OMIM, NetAffyx, UCSC, and Ensemble.

### Chromosomal karyotype analysis

Cytogenetic analysis on long-term cultured amniocytes was performed using standard cytogenetic techniques according to specimen specific protocols, in accordance with the European Society of Human Genetics (ESHG) and European Cytogenetics Association (E.C.A) guidelines. Chromosomes were aged and banded using G-bands by pancreatin and Giemsa staining technique. To investigate the total number and structure of the chromosomes, twenty metaphase cells were visualized and analyzed by qualified cytogenetics technologist using Zeiss microscope Axioskop2 plus (Zeiss, Jena, Germany) with the assistance of the Metasystems imaging system. Furthermore, to rule out a balanced chromosomal rearrangement, involving chromosome 15, parental chromosome analysis from a stimulated peripheral blood lymphocytes was performed.

### Fluorescence in situ hybridization (FISH)

Fluorescence In Situ Hybridization (FISH) was performed at the Klinikum rechts der Isar laboratories (Munich, Germany). FISH analysis was performed using commercial Vysis sub-telomeric probes for chromosome 15 (D15Z1, D15S936) and 18 (D18S552, VIJyRM2050). All specimen types were subjected to standard FISH pretreatment, hybridization, and fluorescence microscopy according to the manufacturer’s specifications and standard specimen specific laboratory protocols. The results of genetic testing were described and reported in accordance with the International Standing Committee on Human Cytogenetic Nomenclature [11].

## Results

### Noninvasive prenatal testing

Professional and detailed genetic counseling regarding the NIPT screening, purpose, significance, accuracy, limitations, and other screening and diagnostic testing options was provided. Informed consent for genetic testing was obtained. A duplication of 10.34 Mb of the long arm of chromosome 15, specifically 15q26.1-q26.3 was reported by noninvasive prenatal testing as an incidental finding. Reported fetal cfDNA was 10.66%. Amniocentesis was performed under the guidance of ultrasound, where approximately 18 ml of amniotic fluid was withdrawn by syringe for diagnostic studies.

### Microarray analysis

**Fig. 1:**
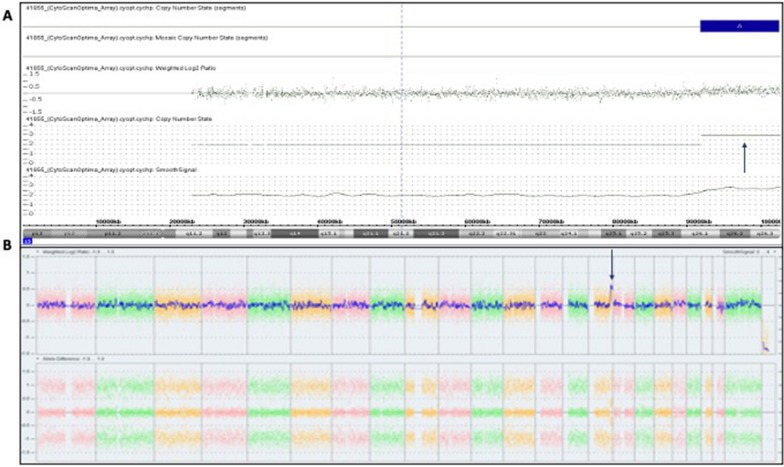
Microarray profile depicting gain (partial trisomy) of the distal long arm of chromosome 15. Representative microarray profile of the fetus showing copy number state and Log2 ratio (**A**-top panel) and the whole genome view (**B**-lower panel) are shown for chromosome 15 (arrows)

The results of the chromosomal microarray analysis confirmed the presence of a pathogenic 10.66 Mb gain of the distal end of chromosome 15, chromosomal region 15q26.1q26.3. More specifically, the duplicated segment includes the chromosomal region between bases 91,763,147 and 102,429,112, encompassing 22 OMIM genes (NCBI Build 37/hg19) (Fig. 1A and B). No other copy number variants were detected, using laboratory’s evaluation criteria. To further characterize this observation, additional studies were completed using G-banding analysis.

### Amniotic fluid karyotype analysis

**Fig. 2:**
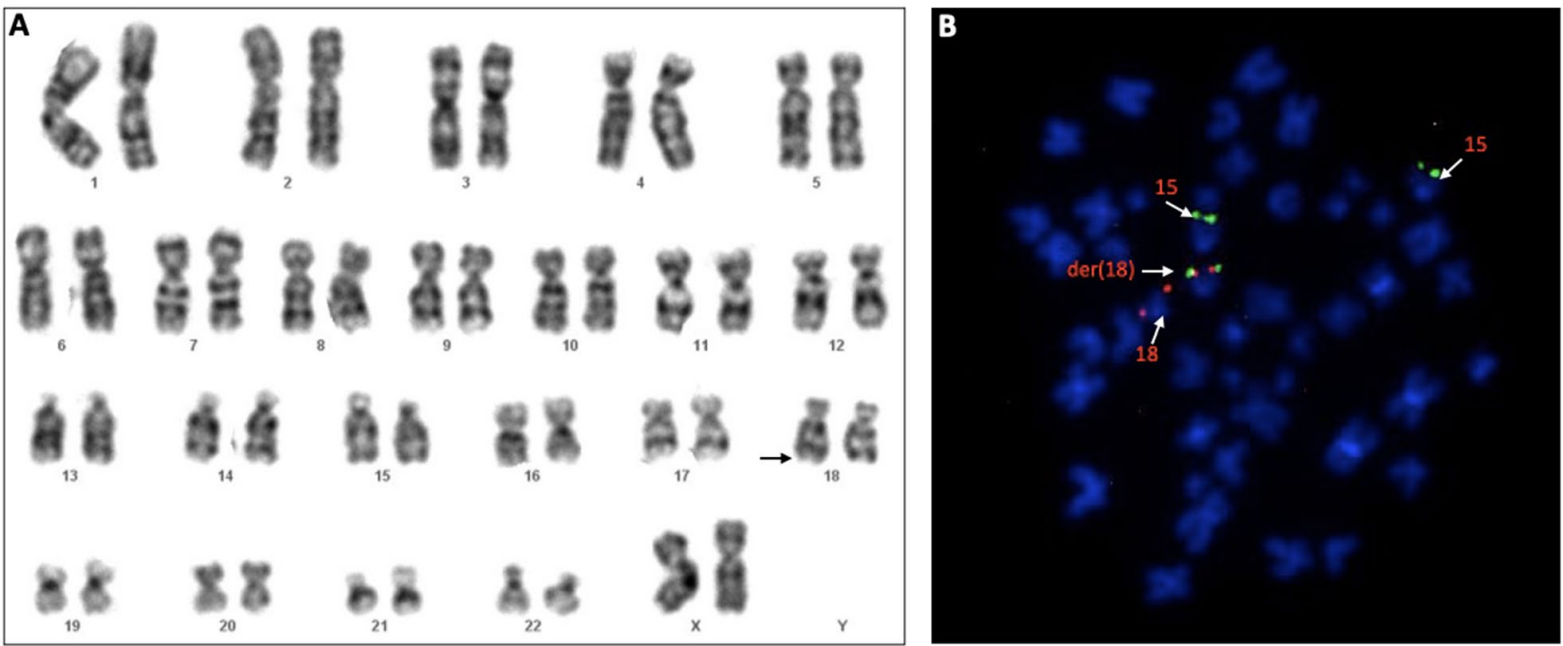
**A** Amniotic fluid karyotype analysis showing additional genetic material on the distal end of the long arm of chromosome 18q23 (arrow). **B** Sequential metaphase FISH analysis utilizing sub-telomeric probes for chromosome 15 (green) and chromosome 18 (red), showing three green signals: two on the normal 15q, and the yellow signal representing a single fusion located on the distal long arm of chromosome 18

Upon analysis of G-banded karyotype obtained from amniotic fluid, it was determined that chromosome 15 does not contain any obvious abnormalities. However, the banding pattern on the long arm of chromosome 18 had an atypical appearance and was suggestive of additional chromatin present on the distal end of the long arm. These findings indicated that the additional chromatin probably originated from the long arm of chromosome 15 (Fig. 2A). The chromosomal morphology/banding of the cells was compromised, precluding our ability to fully characterize the abnormalities present. In an attempt to better characterize structural abnormalities involving chromosomes 15 and 18 observed in the G-banded karyotype analysis, additional studies were completed using FISH techniques (Fig. 2B).

### Amniotic fluid FISH analysis

FISH analysis was performed using sub-telomeric probes for the long arm of chromosomes 15 (green signal) and 18 (red signal). Following hybridization, the probe signal pattern noted was three signals (trisomic imbalance) for chromosome 15q and two signals for chromosome 18q. Two green signals localized to anticipated chromosome 15q region, the third signal being localized to structurally abnormal chromosome 18q, resulting in an unbalanced rearrangement indicated by a single fusion signal (Fig. 2B).

Studies for sub-telomeric region of the long arm of chromosome 18, showed two probe signals and the probes were localized to their anticipated 18q sub-telomeric bands. Thus, the FISH studies provided no evidence for a rearrangement or loss of the sub-telomeric region of chromosome 18. The FISH test confirmed the characterization of the abnormalities involving chromosomes 15 and 18 that were noted in both the microarray and G-banding analyses.

### Parental karyotype analysis

**Fig. 3:**
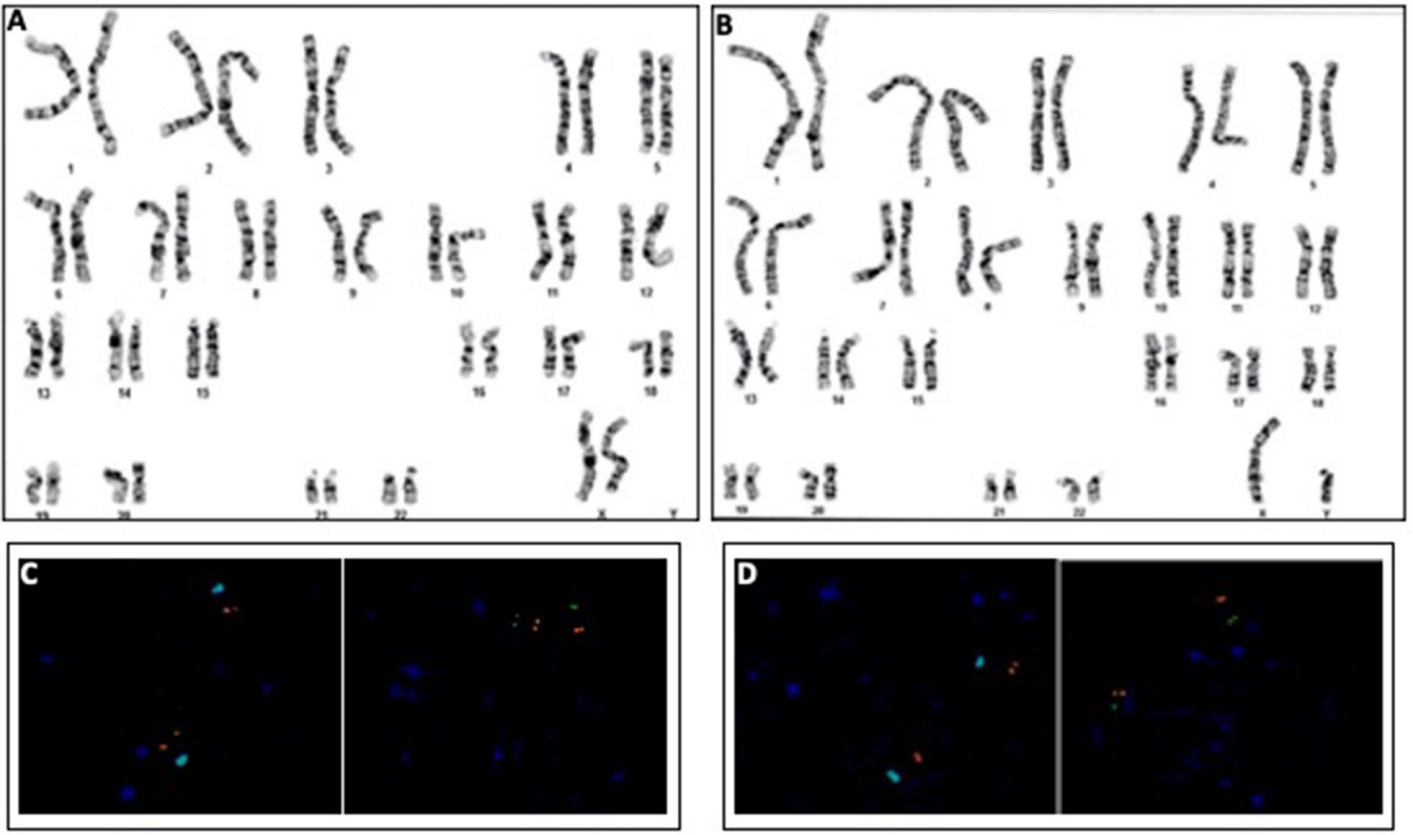
**A** Parental phytohemagglutinin (PHA) stimulated cultured peripheral blood karyograms showing normal karyotypes: A. Maternal karyotype: 46,XX. **B** Paternal karyotype: 46,XY. **C** (maternal) and **D** (paternal). Metaphase FISH analysis showing the presence of the expected number of probes present and the probes were localized to their anticipated regions: two aqua signals for centromeric region (D15Z1) and two orange signals for sub-telomeric region (D15S936) of chromosome 15; two green signals for 18p sub-telomeric probe (D18S552) and two orange signals for 18q sub-telomeric region (VIJyRM2050)

No structural or numerical chromosomal anomalies were detected in parental chromosome analysis (Fig. 3 A and B).

### Parental FISH analysis

Parental metaphase FISH analysis showed the expected number of probes (2 signals for each probe) present, and the probes were localized to their anticipated sub-telomeric bands. There was no evidence for loss or gain of the p or q subtelomeric regions for the chromosomal regions evaluated. Thus, parental FISH studies provided no evidence for a rearrangement between chromosome 15 and chromosome 18 (Fig. 3 C and D).

## Discussion

Because of improvements in the safety of invasive procedures and advances in technology, the most recent systematic reviews demonstrated pregnancy loss rates for CVS and amniocentesis to be less than 1.0% [2, 12]. Naturally, parents-to-be are anxious to have results as early as possible. Cell-free DNA screening takes the advantage of being able to be performed as early as 10 weeks, without the need of an invasive procedure. Noninvasive prenatal screening test has sensitivities and specificities approaching 99%. While NIPT detects aneuploidies with a high degree of certainty, it is, so far, less reliable in detecting microdeletions and duplications in fetal genomes smaller than 5 Mb [13, 14]. Recently, large number of validation studies reporting the PPV of NIPT for the detection of CNVs have been published. Gou et al. demonstrated that the PPV of recurrent CNVs seemed to be higher than that of rare chromosomal deletions/duplications [15]. Furthermore, a recent study by Rafalko et al. reported higher PPV for complex CNVs (93.9%) compared to isolated CNVs (61.0%) [16].

A concern is that as higher proportions of the genome are analyzed, false positive and false negative results are expected to increase, which would result in an increase in unnecessary invasive procedures [17]. NIPT is still a screening test. During pre-test counseling, women should be informed about the accuracy, reliability, false positive, and false negative rates. According to current NIPT guidelines, ACMG strongly recommends all positive NIPT findings to be confirm by invasive prenatal diagnostic testing [17]. In addition, diagnostic follow up testing with CMA should be offered when NIPT identifies a CNV [17].

Partial trisomy of the distal 15q is a rare chromosomal disorder. In general, the duplication of 15q has been characterized by prenatal and postnatal overgrowth, craniosynostosis, distinct facial features, and intellectual disability, likely reflecting triplosensitivity for one or more of the several genes that are found within this region [18–20]. The breakpoints and extent of the duplicated segment are variable among patients. The clinical outcome and severity of physical findings varies from case to case, depending on the length and the genes involved in the duplicated region of the chromosome [19, 21]. Specifically, duplication of type 1 insulin-like growth factor 1 receptor (IGF1R) (OMIM 147,370) gene located at 15q26.3 is thought to lead to overgrowth, whereas haploinsufficiency of IGF1R can cause growth restriction [19–22]. The majority of cases reported have resulted from de novo unbalanced translocations, and the second chromosome involved in the translocation has varied. Although many large duplications can be appreciated by routine karyotyping, detection of this duplication generally requires analysis by fluorescence in situ hybridization and chromosomal microarray. Abnormalities involving sub-telomeric regions can be difficult to visualize well using conventional cytogenetics methods (G-banding analysis).

Previous study has detected the deletion of 5 Mb in fetal chromosome 15q11.2q13.1 and was further confirmed by CNV and karyotype analysis [14]. Our study reports a duplication of about 10 Mb in size detected using NIPT. A finding of duplication of a 10.34 Mb fragment located on 15q26.1q26.3 by NIPT was confirmed using various genetic modalities. The existence of duplication of material from chromosome 15 was established and further delineated by microarray, followed by the karyotype analysis and FISH. Detection of microdeletions or duplications can be very difficult using G-banding karyotyping analysis from amniotic fluid. Therefore, to pinpoint the exact location of the duplicated fragment and to visualize the whole chromosomal complement, subtelomeric probes for the distal portion of chromosomes 15q and 18q were used. The final karyotype from amniotic fluid was reported as 46,XX,der(18)t(15;18)(q26.1;q23)dn.

Without molecular cytogenetic testing modalities, a dup(15)(q26.1q26.3) would have been suggested, but a derivative chromosome 18 would not have been detected. CNV analyses provide information whether copy number gains and losses are present but not whether they have been translocated from their normal position(s) in the genome. Thus, confirmation of suspected chromosome abnormalities by FISH and chromosome analysis may be necessary to determine the nature of an abnormality.

The pregnancy reported in this paper ended in an elective interruption of pregnancy. Since unbalanced translocations can result from either de novo event or the malsegregation of a balanced parental translocation, parental chromosomal studies were completed to see if either of them is a balanced carrier of a translocation involving chromosome 15 and 18. Due to lack of precise and targeted genomic testing done in Bosnia and Herzegovina, diagnostic follow up testing was done in collaboration with laboratories in Greece and Germany. Parental karyotype and FISH analysis were normal with no genetic aberrations. The couple has another healthy child with unknown karyotype. The de novo origin of the 15q duplication is consistent with the fact that there is no prior history of spontaneous abortions and no family history of chromosomal anomalies or genetic disorders. Moreover, it is important to note that negative results in both parents cannot exclude the possibility of parental gonadal mosaicism, thus leading to minor recurrence risk in subsequent pregnancies.

## Conclusion

In conclusion, this case report shows that the resolution of current NIPT technology has the potential to detect submicroscopic aberrations. The clinical application of NIPT screening can be second best choice when patients refuse invasive diagnostic testing, especially for those with no ultrasound findings and no family history. It is clear that NIPT can potentially detect clinically significant unbalanced chromosomal abnormalities present in approximately 1.7% of all structurally normal pregnancies [23]. There is a potentially high value in microdeletion/microduplication NIPT testing mainly for CNVs with substantial morbidity and mortality such as 22q11.2 syndrome. With the typical ultrasound and screening testing many of these cases can go unnoticed or detected late in pregnancy. In these terms NIPT screening could offer an early diagnosis, pregnancy intervention, and coordinated neonatal management. It is a fact that NIPT screening is still in its infancy, facing a lot of challenges and in need of further validation, but such difficulties are expected in the introduction of any new prenatal screening test. Calculation of the test specifications (sensitivity, specificity, PPV, NPV) for submicroscopic imbalances are limited due to lack of confirmatory genetic testing and the prevalence of the disease. Having in mind that all screening tests have to balance between medical benefits and the burden of a false-positive or uncertain finding, NIPT could evolve in another complementary screening tool for early detection of many known syndromes.

## Data Availability

Data sharing is not applicable to this article as no datasets were generated or analyzed during the current study.

## Abbreviations

NIPT: Noninvasive prenatal test
FISH: Fluorescence in situ hybridization
CVS: Chorionic villus sampling
QF-PCR: Quantitative fluorescence polymerase chain reaction
CMA: Chromosomal microarray
NGS: Next-generation sequencing
CNV: Copy number variations
LOH: Loss of heterozygosity
ChAS: Chromosome Analysis Suite
ESGH: European Society of Human Genetics
E.C.A.: European Cytogenetics Association
cfDNA: Cell-free DNA
ACMG: American College of Medical Genetics
IGF1R: Insulin-like growth factor 1 receptor
OMIM: Online Mendelian Inheritance in Man
PPV: Positive predicted value
NPV: Negative predicted value
